# Диагностические возможности двустороннего селективного забора крови из нижних каменистых синусов в различных модификациях и методов лучевой и радионуклидной визуализации в диагностике и дифференциальной диагностике АКТГ-зависимого гиперкортицизма

**DOI:** 10.14341/probl13299

**Published:** 2024-01-24

**Authors:** Ж. Е. Белая, О. О. Голоунина, И. И. Ситкин, Л. Я. Рожинская, М. В. Дегтярев, Д. А. Трухина, Е. В. Бондаренко, А. М. Лапшина, Е. О. Мамедова, Е. Г. Пржиялковская, В. В. Вакс, Г. А. Мельниченко, Н. Г. Мокрышева, И. И. Дедов

**Affiliations:** Национальный медицинский исследовательский центр эндокринологии; Национальный медицинский исследовательский центр эндокринологии; Национальный медицинский исследовательский центр эндокринологии; Национальный медицинский исследовательский центр эндокринологии; Национальный медицинский исследовательский центр эндокринологии; Национальный медицинский исследовательский центр эндокринологии; Национальный медицинский исследовательский центр эндокринологии; Национальный медицинский исследовательский центр эндокринологии; Национальный медицинский исследовательский центр эндокринологии; Национальный медицинский исследовательский центр эндокринологии; Great Western Hospitals NHS Foundation Trust; Национальный медицинский исследовательский центр эндокринологии; Национальный медицинский исследовательский центр эндокринологии; Национальный медицинский исследовательский центр эндокринологии

**Keywords:** болезнь Иценко–Кушинга (БИК), АКТГ-эктопированный синдром, нейроэндокринная опухоль (НЭО), гиперкортицизм, селективный забор крови, мультиспиральная компьютерная томография (МСКТ), соматостатин-рецепторная сцинтиграфия, ОФЭКТ/КТ

## Abstract

**ЦЕЛЬ:**

ЦЕЛЬ. Проанализировать диагностические возможности метода двустороннего селективного забора крови из нижних каменистых синусов (НКС) в различных модификациях в дифференциальной диагностике АКТГ-зависимого эндогенного гиперкортицизма, а также определить чувствительность и специфичность методов топической диагностики в визуализации АКТГ-продуцирующих нейроэндокринных опухолей (НЭО).

**МАТЕРИАЛЫ И МЕТОДЫ:**

МАТЕРИАЛЫ И МЕТОДЫ. Проведено одноцентровое одномоментное диагностическое исследование с ретроспективным анализом данных. В исследование включены пациенты с АКТГ-зависимым эндогенным гиперкортицизмом без визуализации аденомы гипофиза на МРТ или с аденомами размерами менее 6 мм. Всем пациентам выполнен селективный забор крови из НКС с введением десмопрессина в качестве стимуляционного агента, оценкой градиента пролактина и расчетом АКТГ/пролактин-нормализованного отношения или без него. Топическая диагностика АКТГ-продуцирующей опухоли включала проведение МРТ гипофиза, мультиспиральной компьютерной томографии (МСКТ) внутренних органов с контрастным усилением и/или планарной сцинтиграфии и однофотонной эмиссионной компьютерной томографии, совмещенной с компьютерной томографией (ОФЭКТ/КТ) с 99mTc-тектротидом. Диагноз верифицировался по результатам иммуногистохимического исследования или достижения ремиссии болезни Иценко–Кушинга (БИК) после нейрохирургического лечения. Статистическая обработка данных осуществлялась при помощи пакета статистических программ IBM SPSS Statistics 23 (SPSS. Inc, Chicago, IL, USA). Доверительные интервалы рассчитывались с помощью онлайн-калькулятора JavaStat.

**РЕЗУЛЬТАТЫ:**

РЕЗУЛЬТАТЫ. Выполнено 230 селективных заборов у 228 пациентов (166 женщин, 62 мужчин), верифицировано 178 больных с БИК и 50 — с АКТГ-эктопированным синдромом. Результативность катетеризации НКС составила 96,9%. Чувствительность селективного забора крови из НКС без расчета АКТГ/пролактин-нормализованного отношения (n=70) составила 95,9% (95% ДИ 86,3–98,9), специфичность — 92% (95% ДИ 75,0–97,8), для метода селективного забора крови с дополнительным определением АКТГ/пролактин-нормализованного отношения (n=51) — 97,3% (95% ДИ 86,2–99,5) и 93,8% (95% ДИ 71,7–98,9) соответственно. Применение метода МРТ для данной выборки пациентов в верификации БИК имеет чувствительность 60,2% (95% ДИ 52,6–67,5) и специфичность 59,2% (95% ДИ 44,2–73,0). МСКТ имеет чувствительность 74% (95% ДИ 59,7–85,4), специфичность 100% (95% ДИ 97,95–100). Для сцинтиграфии с ОФЭКТ/КТ в визуализации АКТГ-продуцирующих НЭО чувствительность составила 73,3% (95% ДИ 44,9–92,2), специфичность — 100% (95% ДИ 95,3–100).

**ЗАКЛЮЧЕНИЕ:**

ЗАКЛЮЧЕНИЕ. Метод двустороннего селективного забора крови из НКС со стимуляцией десмопрессином и определением концентрации пролактина для контроля положения катетера, а также дополнительным расчетом АКТГ/ пролактин-нормализованного отношения является оптимальным методом дифференциальной диагностики АКТГ-зависимого эндогенного гиперкортицизма. Пациенты с верифицированным АКТГ-эктопированным синдромом по результатам селективного забора крови должны быть дополнительно направлены на сцинтиграфию с ОФЭКТ/КТ с 99mTc-тектротидом и МСКТ для установления локализации НЭО.

## Abstract (en)

**AIM:**

AIM: To analyze the diagnostic performance of bilateral inferior petrosal sinus sampling (BIPSS) with desmopressin as a stimulation agent and prolactin measurements to control catheter position with or without the ACTH/prolactin normalized ratio calculation in the differential diagnosis of ACTH-dependent endogenous hypercortisolism, and the diagnostics performance of ectopic ACTH-syndrome (EAS) visualization.

**MATERIALS AND METHODS:**

MATERIALS AND METHODS: A single-center diagnostic study with a retrospective analysis of the data was carried out. The study included patients with ACTH-dependent endogenous hypercorticism with no visualization of pituitary adenoma on MRI or adenoma sizes less than 6 mm. All patients underwent BIPSS with and without calculation of the ACTH/prolactin normalized ratio. Visualization of an EAS included pituitary MRI (to exclude EAS), whole-body CT scan with contrast, and somatostatin receptor scintigraphy with 99mTc-Tectrotide and CT (99mTc-Tectrotide SPECT). The final verification was based on immunohistochemical confirmation of the tumor or stable remission of Cushing’s disease (CD) after surgical treatment. Statistical data processing was carried out by using IBM SPSS Statistics 23. Confidence intervals were calculated using the JavaStat online calculator.

**RESULTS:**

RESULTS: 230 BIPSS were performed in 228 patients (166 women, 62 men), of which 178 patients were verified as CD and 50 cases were EAS of various localization. The effectiveness of catheterization of petrosal sinuses was 96.9%. The sensitivity of BIPSS without ACTH/prolactin ratio calculation (n=70) was 95.9% (95% CI 86.3–98.9), specificity was 92% (95% CI 75.0–97.8), for the BIPSS with additional determination of ACTH/prolactin-normalized ratio (n=51) — 97.3% (95% CI 86.2–99.5) and 93.8% (95% CI 71.7–98.9), respectively. The use of the MRI method for this sample of patients had a sensitivity of 60.2% (95% CI 52.6–67.5), specificity of 59.2% (95% CI 44.2–73.0), the total body CT with contrast has a sensitivity of 74% (95% CI 59.7–85.4), specificity of 100% (95% CI 97.95–100). The diagnostic accuracy for 99mTc-Tectrotide SPECT in NET visualization has a sensitivity of 73.3% (95% CI 44.9–92.2), specificity of 100% (95% CI 95.3–100).

**CONCLUSION:**

CONCLUSION: BIPSS with desmopressin stimulation and prolactin measurements to control catheter position, as well as the additional calculation of the ACTH/prolactin-normalized ratio, is an optimal method for the differential diagnosis of EAS. Patients who are identified an EAS on BIPSS may be further referred for 99mTc-Tectrotide SPECT and CT for tumor visualization.

## ОБОСНОВАНИЕ

Эндогенный гиперкортицизм — одно из наиболее тяжелых эндокринных заболеваний, клинические проявления которого обусловлены длительным воздействием на организм избыточного количества кортизола [1]. В 80% случаев причина заболевания — АКТГ-секретирующая аденома гипофиза (болезнь Иценко–Кушинга (БИК)). Значительно более редким вариантом АКТГ-зависимого гиперкортицизма является АКТГ-эктопированный синдром, обусловленный избыточной продукцией АКТГ, реже кортикотропин-рилизинг-гормона (КРГ), нейроэндокринной опухолью (НЭО) различной локализации. Бронхолегочные карциноиды диагностируются примерно в 61% случаев, в 11% источником заболевания являются НЭО средостения [2]. АКТГ-секретирующие НЭО могут локализоваться в желудочно-кишечном тракте, поджелудочной железе, почке. Эктопическая продукция АКТГ обнаруживается также при опухолях эндокринных желез, например при феохромоцитоме и медуллярном раке щитовидной железы [3].

Определенную сложность представляет дифференциальная диагностика АКТГ-зависимых форм эндогенного гиперкортицизма. В первую линию диагностического поиска АКТГ-продуцирующих опухолей обычно входит такой метод исследования, как магнитно-резонансная томография (МРТ). МРТ остается ведущим методом лучевой диагностики опухолей гипофиза, однако по-прежнему имеет недостаточную чувствительность для выявления АКТГ-продуцирующих микроаденом. Кроме того, до 20% населения могут быть носителями гормонально-неактивных аденом гипофиза [4]. Таким образом, выявление микроаденомы гипофиза, особенно менее 5–6 мм в диаметре, необязательно означает, что найдена причина эндогенного гиперкортицизма, а пациенты с АКТГ-эктопированным синдромом нередко могут иметь опухоль гипофиза в сочетании с карциноидной опухолью другой локализации [3].

Консенсус по диагностике и ведению пациентов с БИК, а также российские клинические рекомендации рекомендуют при наличии образования гипофиза 6–9 мм и более устанавливать диагноз БИК, не требуя проведения дополнительных тестов [5][6]. Точность порогового значения размера опухоли 6 мм в дифференциальной диагностике БИК и АКТГ-эктопированного синдрома изучена в работе Yogi-Morren D. и соавт. [7]. В исследование включены 130 пациентов, из них у 104 больных верифицирована БИК, у 26 — АКТГ-эктопированный синдром. Аденома гипофиза визуализировалась у 71/104 (68,3%) и 6/26 (23%) пациентов со средними размерами образования 8 мм (2–31 мм) и 5 мм (3–14 мм) соответственно. Наличие опухоли гипофиза более 6 мм имело чувствительность 40% и специфичность 96% в диагностике БИК, то есть всего 4% пациентов с аденомами более 6 мм имели эктопическую продукцию АКТГ.

В последнее время ведется активный поиск надежных биомаркеров, позволяющих проводить минимально инвазивную раннюю диагностику и дифференциальную диагностику эндогенного гиперкортицизма и его АКТГ-зависимых форм. Так, проведен ряд исследований оценки уровня экспрессии микроРНК в плазме крови пациентов с АКТГ-зависимым эндогенным гиперкортицизмом, в том числе выявлены различно экспрессирующиеся циркулирующие микроРНК в плазме крови у пациентов с БИК и АКТГ-эктопированным синдромом [8][9].

Другим перспективным подходом для ранней дифференциальной диагностики может являться изучение опухолеспецифических различий в процессинге, синтезе и экспрессии различных пептидов, их предшественников и других низкомолекулярных непептидных соединений, которые возможно исследовать и выявить с помощью протеомного анализа. Ранние исследования продемонстрировали высокие концентрации предшественников АКТГ в плазме у пациентов с АКТГ-эктопированным синдромом, свидетельствующие о том, что в АКТГ-­продуцирующих НЭО проопиомеланокортин, предшественник АКТГ, не подвергается нормальному посттрансляционному процессингу [10].

Параллельно совершенствуются неинвазивные методы визуализации АКТГ-продуцирующих опухолей, такие как мультиспиральная компьютерная томографии (МСКТ), позволяющая увидеть различные по плотности структуры, планарная сцинтиграфия, однофотонная эмиссионная компьютерная томография, совмещенная с компьютерной томографией (ОФЭКТ/КТ), которая дает полное представление об анатомическом расположении опухоли и рецепторной плотности, совмещенная позитронно-эмиссионная и компьютерная томография (ПЭТ/КТ), дающая информацию о метаболизме, функциональной активности образований [11]. Чувствительность и специфичность всех доступных методов диагностики напрямую зависят от квалификации специалиста, проводящего исследование, технических характеристик оборудования и, следовательно, сильно варьируют между медицинскими центрами. Несмотря на большое количество доступных современных методов лучевой и радионуклидной диагностики, неоднократно проведенные исследования, зачастую не удается выявить локализацию опухоли. Необходимо отметить, что топический поиск источника эктопической продукции АКТГ оправданно проводить только после верификации диагноза по результатам селективного забора крови из нижних каменистых синусов (НКС) ввиду того, что определение локализации опухоли до проведения селективного забора обладает низкой чувствительностью и специфичностью.

На сегодняшний день метод двустороннего селективного забора крови из НКС с определением градиента АКТГ центр/периферия до и после введения стимуляционного агента признан «золотым стандартом» среди всех методов дифференциальной диагностики АКТГ-зависимых форм эндогенного гиперкортицизма [12][13]. С момента внедрения метод претерпел значительные изменения и усовершенствования с целью повышения диагностической точности. Так, помимо стимуляционного агента проводится контроль положения катетеров с определением градиента пролактина, применяются различные стимуляционные агенты (кортиколиберин, десмопрессин и их комбинация), расчет АКТГ/пролактин-нормализованного отношения, а также комбинация селективного забора крови из НКС с гибридными методами функциональной и топической визуализации [14][15].

## ЦЕЛЬ ИССЛЕДОВАНИЯ

Цель исследования — проанализировать диагностические возможности двустороннего селективного забора крови из НКС в различных модификациях в дифференциальной диагностике АКТГ-зависимых форм эндогенного гиперкортицизма, а также определить чувствительность и специфичность различных методов топической диагностики в визуализации АКТГ-продуцирующих НЭО.

## МАТЕРИАЛЫ И МЕТОДЫ

## Место и время проведения исследования

Место проведения. Исследование проведено на базе отделения нейроэндокринологии и остеопатий ГНЦ РФ ФГБУ «НМИЦ эндокринологии» Минздрава России.

Время исследования. В исследование включены пациенты, находившиеся на стационарном обследовании и лечении в указанном выше отделении в период с 28.10.2015 по 29.12.2022 гг.

## Изучаемые популяции

Пациенты с подтвержденным диагнозом АКТГ-зависимого эндогенного гиперкортицизма.

Критерии включения: лабораторно подтвержденный АКТГ-зависимый эндогенный гиперкортицизм: АКТГ>10 нг/мл в сочетании с повышенным уровнем кортизола в суточной моче и другими тестами, подтверждающими наличие эндогенного гиперкортицизма (повышение кортизола в вечерней слюне, и/или отрицательная малая дексаметазоновая проба, и/или повышение уровня кортизола в вечернее время); отсутствие визуализации аденомы гипофиза на МРТ; или размеры аденомы гипофиза менее 6 мм; или отрицательная большая дексаметазоновая проба; или отсутствие ремиссии после предыдущей нейрохирургической операции, когда послеоперационный материал недоступен для иммуногистохимического подтверждения диагноза или неинформативен.

Критерии невключения: двусторонняя адреналэктомия в анамнезе; прием препаратов для консервативного лечения гиперкортицизма менее чем за 2 нед до участия в исследовании (каберголин, пасиреотид, октреотид короткого действия, кетоконазол); терапия аналогами соматостатина пролонгированного действия менее чем за 4 нед до участия в исследовании; хроническая болезнь почек (СКФ<30 мл/мин); повышенная чувствительность к контрастным препаратам и/или к любому компоненту радиофармпрепарата (РФП); тяжелые нарушения гомеостаза; период беременности, родов, период грудного вскармливания; наличие психических заболеваний.

## Способ формирования выборки из изучаемой популяции

Способ формирования выборки — сплошной, в соответствии с критериями включения.

Согласно полученным в ходе селективного забора крови из НКС результатам, все пациенты разделены на две группы: пациенты с БИК (группа 1) и пациенты с АКТГ-эктопированным синдромом (группа 2). Критерий «золотого стандарта» — иммуногистохимическая верификация опухоли или наступление стойкой ремиссии после операции, если опухоль недоступна.

## Дизайн исследования

Одноцентровое одномоментное диагностическое исследование с ретроспективным анализом данных.

## Описание медицинского вмешательства (для интервенционных исследований)

У всех больных, включенных в исследование, на этапе скрининга лабораторно и клинически подтвержден АКТГ-зависимый эндогенный гиперкортицизм в соответствии с клиническими рекомендациями [6] и определены показания к проведению селективного забора крови из НКС. Перед вмешательством пациенты осмотрены анестезиологом, по показаниям — кардиологом.

Всем пациентам, включенным в исследование, выполнялся селективный забор крови из НКС на фоне стимуляции десмопрессином (Desmopressin Acetate 4 мкг в 1 мл для в/в, в/м и п/к инъекций Ferring Pharmaceuticals) в дозе 8 мкг, по стандартной методике, с использованием одностороннего венозного трансфеморального доступа. Во всех случаях интервенция проводилась под местной анестезией. В ходе вмешательства выполнялся мониторинг артериального давления, пульса, электрокардиограммы.

Забор венозной крови из правого, левого НКС и нижней полой вены проводился одномоментно с экспозицией в 5 мин до введения десмопрессина в/в и 3 раза с интервалом 3, 5 и 10 мин после введения десмопрессина. В указанных интервалах времени кровь исследовалась на АКТГ. Лабораторная оценка концентрации уровня пролактина и АКТГ в венозной крови из НКС относительно концентрации этих гормонов в периферической пробе использовалась для контроля положения катетера. Градиент пролактина рассчитывали как отношение максимального уровня пролактина из центрального образца крови (ипсилатеральный максимальному уровню АКТГ) к уровню пролактина в периферической вене. Градиент пролактина ≥1,5 свидетельствовал об адекватной установке катетера. АКТГ/пролактин-нормализованное отношение рассчитывали как отношение максимального градиента АКТГ после введения десмопрессина к ипсилатеральному градиенту базального пролактина. Градиент более 1,18 свидетельствовал в пользу БИК [14]. Результаты забора крови оценивались на основании расчетного максимального отношения уровней АКТГ в синусах к периферическому уровню АКТГ до стимуляции десмопрессином и после введения препарата внутривенно. Градиент АКТГ центр/периферия ≥2 до стимуляции и/или ≥3 после стимуляции десмопрессином свидетельствовал в пользу БИК, более низкие значения градиента АКТГ центр/периферия расценивались как АКТГ-эктопированный синдром.

При подтверждении диагноза БИК по результатам селективного забора крови пациент направлялся для проведения нейрохирургического лечения в объеме трансназальной транссфеноидальной аденомэктомии с применением эндоскопических технологий (при отсутствии противопоказаний и наличии согласия больного). При подтверждении АКТГ-эктопированного синдрома проводился диагностический поиск источника гиперпродукции АКТГ, и в случае выявления НЭО пациент направлялся на соответствующее хирургическое лечение в специализированное медицинское учреждение.

## Методы

МРТ головного мозга проводилась на магнитно-резонансном томографе Magnetom Harmony (Siemens, Германия) с напряженностью поля от 1,5 до 3 Тесла с введением гадолиниевого контрастного препарата.

Поиск новообразования, секретирующего АКТГ, включал проведение МСКТ органов грудной клетки, средостения, органов брюшной полости и забрюшинного пространства с внутривенным болюсным контрастированием. Исследование проводилось на мультидетекторном компьютерном томографе Optima CT фирмы General Electric. Толщина срезов при исследовании составляла 0,625 мм.

Планарная сцинтиграфия и ОФЭКТ/КТ с РФП (99mTc-EDDA/HYNIC-TOC (99mTc-тектротид)) выполнены на томографе ОФЭКТ/КТ GE Discovery NM/CT 670 с использованием низкоэнергетических коллиматоров высокого разрешения (LEHR) в режиме сканирования «всего тела». На первом этапе для оценки накопления и распределения РФП выполнялась планарная сцинтиграфия в режиме сканирования «всего тела». Затем с целью анатомической визуализации очагов патологического накопления РФП проводилась ОФЭКТ/КТ одной или более зон интереса. Обработка результатов сцинтиграфии и ОФЭКТ/КТ осуществлялась на рабочей станции радиолога. Оценка планарной сцинтиграфии выполнялась стандартными приемами: сглаживание (фильтрация), контрастирование (изменение градиентной шкалы), качественное сравнение симметричных зон интереса. Визуализация очагов патологического, повышенного накопления РФП вне зон его физиологической аккумуляции (печень, селезенка, почки, мочевой пузырь, кишечник) свидетельствовали о наличии активной опухолевой ткани соответственно их локализации. Гормональное исследование АКТГ (референсный интервал утро 7,2–63,3 пг/мл, вечер 2–25,5 пг/мл), кортизола в сыворотке крови в 23:00 (64–327 нмоль/л), определение свободного кортизола в вечерней слюне (0,5–9,6 нмоль/л) проводилось электрохемилюминесцентным методом на анализаторе Cobas 6000 Module e601 (Roche); определение содержание пролактина в сыворотке крови, измерение свободного кортизола в суточной моче (100–379 нмоль/сут) — иммунохемилюминесцентным методом на аппарате Vitros ECi.

Для оценки эффективности трансназальной аденом­эктомии или резекции НЭО в раннем послеоперационном периоде определялись уровни АКТГ и кортизола крови в 8:00. При снижении концентрации кортизола крови ниже референсных значений пациенту назначалась заместительная терапия глюкокортикоидами. При сомнении в наличии послеоперационной ремиссии заболевания дополнительно проводились анализы на свободный кортизол в суточной моче и/или свободный кортизол в слюне в 23:00.

Окончательный диагноз верифицировался по результатам гистологического и иммуногистохимического исследования с оценкой экспрессии АКТГ в удаленной опухоли. Опухоли гипофиза верифицировали в соответствии с Международной гистологической классификацией опухолей гипофиза (ВОЗ, 2017, ВОЗ 2004), НЭО — в соответствии с органоспецифичными классификациями ВОЗ [16–18].

Иммуногистохимическое исследование проводили в соответствии со стандартным протоколом в автоматическом режиме на срезах толщиной 3 мкм, расположенных на стеклах с полилизиновым слоем (Leica, Германия). В качестве маркеров-антител использовали АКТГ (mouse, клон 02А3), КРГ (rabbit, клон FL-196).

В исключительных случаях при отсутствии или недостаточном количестве опухолевого материала косвенным признаком подтверждения диагноза считали наступление стойкой ремиссии после операции.

## Статистический анализ

Количественные данные представлены в виде средних значений со среднеквадратическим отклонением, медианы (Me) с указанием интерквартильного диапазона [Q25–Q75], максимальных и минимальных значений, качественные переменные представлены в виде абсолютных и относительных частот. Соотношения качественных признаков представлены в виде долей (%). Сравнение двух независимых групп для количественных данных выполнялось с помощью критерия Стьюдента для признаков, соответствующих закону нормального распределения, и критерия Манна–Уитни для признаков, не соответствующих закону нормального распределения. Качественные переменные сравнивались между собой с помощью критерия Хи квадрат (χ²) и точного двустороннего критерия Фишера.

Вычисление основных показателей эффективности диагностического метода, таких как диагностическая чувствительность, диагностическая специфичность, прогностическая ценность положительного результата (количество пациентов, истинно положительных по результатам тестирования/(количество пациентов, истинно положительных при тестировании + количество пациентов, ложно­положительных при тестировании)), общая точность, прогностическая ценность отрицательного результата (количество пациентов с истинно негативным результатом теста/(количество истиннонегативных результатов + количество ложнонегативных результатов)), отношение правдоподобия для положительного результата теста (чувствительность/(1-специфичность)) проводилось по специальным формулам [19]. Площадь под кривыми операционных характеристик была рассчитана для представления возможности теста прогнозировать истинно положительный и истинно отрицательный результаты.

Статистическая обработка данных осуществлялась при помощи пакета статистических программ IBM SPSS Statistics 23 (SPSS. Inc, Chicago, IL, USA). Доверительные интервалы рассчитывались с помощью онлайн-калькулятора JavaStat (https://statpages.info/ctab2x2.html).

## Этическая экспертиза

Протокол исследования одобрен локальным этическим комитетом ГНЦ РФ ФГБУ «НМИЦ эндокринологии» Минздрава России, выписка из протокола №2 от 20.02.2013 г. Все пациенты, включенные в исследование, подписали информированное согласие на участие в исследовании.

## РЕЗУЛЬТАТЫ

## Объекты (участники) исследования

Основу исследования составил анализ результатов двустороннего селективного забора крови из НКС, выполненного 228 пациентам (166 женщин, 62 мужчин) с АКТГ-зависимым эндогенным гиперкортицизмом: у 70 больных селективный забор крови проведен только с контролем положения катетера по градиенту пролактина; у 51 пациента дополнительно рассчитывалось АКТГ/пролактин-нормализованное отношение; 107 пациентам после процедуры селективного забора крови с определением градиента пролактина и АКТГ/пролактин-нормализованного отношения выполнялась соматостатин-рецепторная сцинтиграфия с 99mTc-тектротидом (рис. 1).

**Figure:**
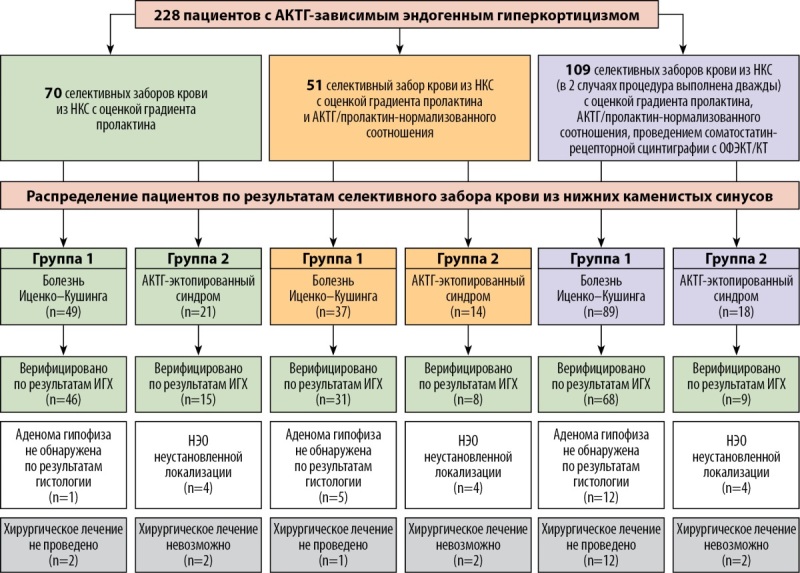
Рисунок 1. Схема-дизайн исследования.Примечание: НКС — нижние каменистые синусы; ИГХ — иммуногистохимическое исследование; НЭО — нейроэндокринная опухоль.

Общая характеристика пациентов, основные гормональные показатели и результаты инструментальных и радионуклидных методов исследований представлены в табл. 1. Все пациенты распределены на две группы по окончательному клиническому диагнозу согласно результатам гистологического исследования. Несмотря на то что уровни АКТГ и кортизола во всех биологических жидкостях в обеих группах значимо различались (p≤0,001 для всех показателей), сложно провести дифференциальную диагностику между БИК и АКТГ-эктопированным синдромом исключительно по концентрации вышеназванных гормонов в связи с разбросом индивидуальных показателей. Проведение большой дексаметазоновой пробы позволило выявить различия в системе регуляции гипоталамо-гипофизарно-надпочечниковой оси между группами, однако у 17,3% пациентов с БИК проба оказалась отрицательной, тогда как в 17,1% случаев у пациентов с АКТГ-эктопированным синдромом после приема 8 мг дексаметазона наблюдалось снижение уровня кортизола на 60% и более от исходного.

**Table:** Таблица 1. Общая характеристика и гормональные показатели включенных в исследование пациентов с АКТГ-зависимым эндогенным гиперкортицизмом. Окончательный диагноз по результатам гистологического исследования

Параметр	Пациенты с болезнью Иценко–Кушинга	Пациенты с АКТГ-эктопированным синдромом	р
Общее количество пациентов	178	50	–
Мужчины (%) : Женщины (%)	42 (23,6%) : 136 (76,4%)	20 (40%) : 30 (60%)	0,030
Возраст на момент проведения селективного забора крови, лет	39 [ 30; 50] (18; 72)	43 [ 31; 56] (18; 76)	0,201
Индекс массы тела, кг/м²	31,2 [ 26,6; 34,7] (18,3; 57,5)	29 [ 25,3; 33,5] (21; 48,7)	0,129
Лабораторные параметры на момент установки диагноза
АКТГ в 08:00, пг/мл	63,6 [ 45,7; 82,2] (13,4; 428,3)	132,3 [ 98,3; 184] (40; 536,7)	<0,001
АКТГ в 23:00, пг/мл	51,8 [ 33,3; 75,5] (8,1; 252,7)	108,6 [ 78; 176,4] (38; 755,6)	<0,001
Кортизол в слюне 23:00, нмоль/л	22,6 [ 14,7; 40,4] (4,2; 436)	60,1 [ 35,4; 117] (9,8; 711,5)	<0,001
Кортизол в суточной моче, нмоль/сут	1198,4 [ 725,6; 2362,6] (284; 15196)	3460,4 [ 1691,2; 6982,6] (640,2; 12332,25)	<0,001
Кортизол в крови в 23:00, нмоль/л	662,6 [ 520,5; 907,6] (93,2; 1960)	1115,5 [ 731,8; 1390,8] (460,2; 5854,8)	<0,001
Малая дексаметазоновая проба
Отрицательная	176	50	0,753
Положительная	1	0
Большая дексаметазоновая проба
Отрицательная	14	26	<0,001
Положительная	67	6
Визуализация аденомы на МРТ
С визуализацией	106	20	0,022
Без визуализации	70	29
МРТ не выполнялось	2	1	–
Визуализация нейроэндокринной опухоли на МСКТ с контрастным усилением
С визуализацией	0	37	–
Без визуализации	0	13	–
Планарная сцинтиграфия, совмещенная с ОФЭКТ/КТ с 99mTc-Тектротидом
Не проводилась	101	35	–
Нейроэндокринная опухоль не выявлена	77	4	–
Нейроэндокринная опухоль выявлена	0	11	–

Из 228 пациентов трем больным не удалось провести визуализацию гипофиза методом МРТ ввиду наличия кардиостимулятора, клаустрофобии, морбидного ожирения (ИМТ=57,5 кг/м²). Выполнение МРТ головного мозга позволило выявить аденому гипофиза у 126 пациентов, при этом патологических изменений в гипофизе при МРТ с внутривенным введением контрастного препарата не обнаружено у 99 больных. В группе пациентов с БИК аденома гипофиза визуализирована в 60,2% случаев (106/176), у 40,8% пациентов (20/49) с АКТГ-эктопированным синдромом также выявлена микроаденома гипофиза по данным МРТ головного мозга, из них 4 пациента первоначально были подвергнуты нейрохирургической операции с предварительным диагнозом БИК, однако впоследствии всем из них установлен диагноз «АКТГ-эктопированный синдром» с локализацией НЭО в легком в 2 случаях и в 2 случаях первичный очаг заболевания до настоящего времени остается неустановленным.

Таким образом, показанием для проведения селективного забора крови из НКС в 99 случаях было отсутствие визуализации аденомы гипофиза на МРТ, в 3 — невозможность проведения МРТ головного мозга и отсутствие данных за НЭО по результатам МСКТ, у 108 пациентов — размер аденомы гипофиза менее 6 мм, у 7 больных аденома гипофиза более 6 мм в сочетании с отрицательной большой дексаметазоновой пробой, у 11 пациентов — визуализация микроаденомы гипофиза менее 6 мм, отсутствие ремиссии после предыдущей нейрохирургической операции и невозможность иммуногистохимического подтверждения диагноза.

## Диагностические возможности селективного забора крови из НКС в дифференциальной диагностике АКТГ-зависимого гиперкортицизма

Для уточнения диагноза 228 пациентам, включенным в исследование, выполнен селективный забор крови из НКС, при этом у 2 больных данная процедура проведена дважды и оба раза была неинформативной в связи с отсутствием концентрационного градиента по пролактину из-за особенностей анатомического оттока крови от гипофиза. Результативность катетеризации НКС составила 96,9%, градиент пролактина был отрицательным у 7 пациентов (3,1%), из них в 6 случаях максимальное отношение АКТГ центр/периферия свидетельствовало в пользу центрального генеза эндогенного гиперкортицизма.

У пациентов с БИК максимальный градиент АКТГ между синусом и периферией до стимуляции составлял 12,02±7,35, после введения десмопрессина — 20,62±14,04. Максимальный градиент АКТГ центр/периферия у пациентов с АКТГ-эктопированным синдромом до и после стимуляции составил 1,20±0,22 и 1,38±0,37 соответственно.

Суммарные характеристики диагностических возможностей метода селективного забора крови из НКС с использованием стимуляционного агента сведены в табл. 2. Максимальное отношение АКТГ центр/периферия до введения десмопрессина позволило верифицировать БИК с чувствительностью 96,07% (95% ДИ 92,11–98,08) и специ­фичностью 88,33% (95% ДИ 77,82–94,23), тогда как введение десмопрессина в дозе 8 мкг увеличило чувствительность до 98,31% (95% ДИ 95,16–99,43) и специфичность до 94,64% (95% ДИ 85,39–98,16). Площадь под ROC-кривой для метода катетеризации НКС без введения десмопрессина составила 0,980 (95% ДИ 0,963–0,997) и была выше при анализе данных после введения десмопрессина — 0,992 (95% ДИ 0,980–1,000).

**Table:** Таблица 2. Суммарные характеристики возможностей одномоментного селективного забора крови из НКС на фоне стимуляции десмопрессином для диагностики БИК среди пациентов с АКТГ-зависимым гиперкортицизмом

Отрезная точка	Максимальное отношение АКТГ центр/периферия ≥2 до введения десмопрессина и/или ≥3 после введения десмопрессина внутривенно
Чувствительность, % (95% ДИ)	98,31 (95,16–99,43)
Специфичность, % (95% ДИ)	94,64 (85,39–98,16)
Прогностическая ценность положительного результата теста (истинно положительный результат) (95% ДИ)	18,35 (6,10–55,19)
Прогностическая ценность отрицательного результата теста (истинно отрицательный результат) (95% ДИ)	0,02 (0,006–0,055)
Отношение правдоподобия положительного результата теста (95% ДИ)	1030,56 (202,01–5247,41)

Осложнения после процедуры селективного забора крови из НКС зарегистрированы в 0,9% случаев (2/228 пациентов): у обеих пациенток на 2-е и 3-и сутки после селективного забора крови развились острый тромбоз и тромбофлебит соответственно большой подкожной вены справа. Других серьезных нежелательных явлений выявлено не было. Таким образом, метод двустороннего селективного забора крови из НКС имеет высокую безопасность и эффективность.

Роль определения уровня пролактина для контроля положения катетера в ходе селективного забора крови из НКС

70 пациентам (47 женщин, 23 мужчины) двусторонний селективный забор крови из НКС проведен с контролем положения катетера по градиенту пролактина. Медиана возраста на момент заболевания составила 34 года [ 19; 44].

В ходе процедуры селективного забора крови из НКС у 49 из 70 пациентов максимальное отношение АКТГ центр/периферия после введения десмопрессина превышало установленную отрезную точку (градиент АКТГ более 3), что свидетельствовало в пользу БИК, при этом исходно до стимуляции у 2 больных градиент АКТГ составил менее 2, но значительно увеличился, в 3,2 и 19,6 раза соответственно, после введения десмопрессина. В отличие от пациентов с БИК, введение десмопрессина больным с АКТГ-эктопированным синдромом практически не увеличивало продукцию АКТГ, а максимальный градиент АКТГ центр/периферия был менее 3, в связи с чем по результатам селективного забора 21 пациенту установлен клинический диагноз «АКТГ-эктопированный синдром».

Градиент пролактина не получен только в 1 случае, таким образом, результативность катетеризации НКС составила 98,6%.

Чувствительность метода селективного забора крови из НКС с введением стимуляционного агента и контролем положения катетера по градиенту пролактина составила 95,9% (95% ДИ 86,3–98,9), специфичность — 92% (95% ДИ 75,0–97,8).

Значение расчета АКТГ/пролактин-нормализованного отношения

Пятьдесят одному пациенту (34 женщины, 17 мужчин) в ходе процедуры селективного забора крови, помимо определения уровня пролактина с расчетом его градиента центр/периферия для контроля положения катетера, дополнительно рассчитывалось АКТГ/пролактин-нормализованное отношение для улучшения диагностических возможностей метода.

Градиент пролактина получен во всех случаях, следовательно, результативность катетеризации НКС составила 100%.

Результат оценки АКТГ/пролактин-нормализованного отношения считался истинно положительным (n=37), когда у пациентов с гистологически подтвержденной БИК АКТГ/пролактин-нормализованное отношение ≥1,18; истинно отрицательным (n=13), если у пациентов с гистологически подтвержденным АКТГ-эктопированным синдромом АКТГ/пролактин-нормализованное отношение <1,18; ложноположительным в случае, если у пациентов с БИК АКТГ/пролактин-нормализованное отношение <1,18, и, наконец, ложноотрицательным (n=1), когда у пациентов с АКТГ-эктопированным синдромом АКТГ/пролактин-нормализованное отношение ≥1,18.

Чувствительность метода селективного забора крови из НКС с использованием стимуляционного агента, контролем положения катетера по градиенту пролактина и определением АКТГ/пролактин-нормализованного отношения составила 97,3% (95% ДИ 86,2–99,5), специфичность — 93,8% (95% ДИ 71,7–98,9). Площадь под кривой операционной характеристики диагностических возможностей селективного забора крови из НКС со стимуляцией десмопрессином и расчетом АКТГ/пролактин-нормализованного отношения составила 0,964 (95% ДИ 0,885–1,000).

Диагностические возможности метода гибридной топической диагностики с проведением селективного забора крови из НКС и соматостатин-рецепторной сцинтиграфии

В анализ включены данные 107 пациентов (85 женщин, 22 мужчины). Медиана возраста на момент заболевания составила 36 лет [ 28; 51].

По результатам селективного забора крови из НКС у 89 пациентов верифицирована БИК, у 18 — АКТГ-эктопированный синдром, однако при анализе данных выявлено, что в 3 случаях получен ложноположительный результат: у 2 больных отсутствовал градиент пролактина несмотря на то, что процедура выполнялась дважды, 1 пациент не имел градиента АКТГ центр/периферия исходно и в ответ на стимуляцию.

В ходе селективного забора крови из НКС градиент пролактина не получен в 6 случаях, таким образом, результативность катетеризации НКС составила 94,4%. При расчете АКТГ/пролактин-нормализованного отношения в 2 случаях получен ложноположительный результат, истинноотрицательный результат — у 13 пациентов, у 2 больных — ложноотрицательный, в остальных случаях получен истинно положительный результат. Таким образом, чувствительность метода селективного забора крови из НКС в данной части исследования составила 95,7% (95% ДИ 89,4–98,3), специфичность — 82,6% (95% ДИ 62,9–93,0).

После верификации формы АКТГ-зависимого эндогенного гиперкортицизма третьим этапом 92 пациентам выполнена соматостатин-рецепторная сцинтиграфия, совмещенная с ОФЭКТ/КТ, с 99mTc-тектротидом, по результатам которой сцинтиграфические признаки гормонально-активного образования (НЭО) с гиперэкспрессией соматостатиновых рецепторов выявлены у 11 пациентов. У 4 больных с внегипофизарной локализацией АКТГ-продуцирующей НЭО первичный опухолевый очаг оставался неустановленным. Нежелательных явлений при применении РФП в рамках соматостатин-рецепторной сцинтиграфии не было зарегистрировано ни у одного пациента. При расчете площади под ROC-кривыми для соматостатин-рецепторной сцинтиграфии, совмещенной с ОФЭКТ/КТ, в отношении топической диагностики АКТГ-продуцирующих опухолей значение составило 0,888 (95% ДИ 0,774–1,000), что несколько выше, чем для МСКТ с контрастным усилением, где площадь под ROC-кривой равна 0,870 (95% ДИ 0,796–0,944) (рис. 2). Среди 50 пациентов с установленным диагнозом «АКТГ-эктопированный синдром» в 32 случаях источником эктопической продукции АКТГ являлись НЭО бронхолегочной локализации, у 3 больных — НЭО, локализованные в надпочечнике, у 2 — НЭО поджелудочной железы, в 1 случае — НЭО средостения, у 12 пациентов первичный опухолевый очаг локализовать не удалось. Минимальный размер образования, выявленного при проведении соматостатин-рецепторной сцинтиграфии, совмещенной с ОФЭКТ/КТ с 99mTc-тектротидом, составил 5×7 мм, максимальный — 43×37×53 мм. Данный метод позволил локализовать 9 НЭО легкого, 1 НЭО надпочечника и 1 НЭО поджелудочной железы. У пациентки с образованием хвоста поджелудочной железы с гиперэкспрессией соматостатиновых ­рецепторов ­дополнительно выявлены ­образования по нижнему контуру тела и несколько краниальнее хвоста поджелудочной железы с признаками накопления 99mTc-Тектротида (вторично измененные лимфатические узлы), а также фокусы гипераккумуляции РФП в печени (mts).

**Figure:**
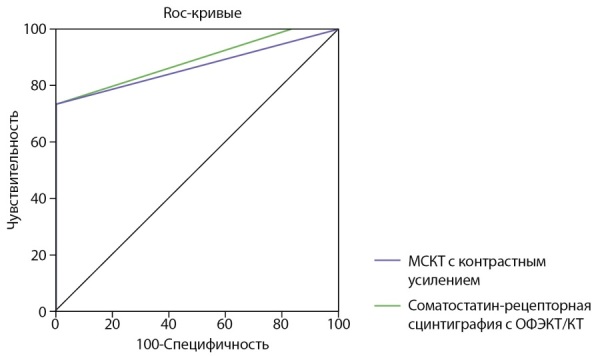
Рисунок 2. Прямое сравнение площадей под кривыми операционных характеристик использования МСКТ с контрастным усилением и соматостатин-рецепторной сцинтиграфии с ОФЭКТ/КТ для топической диагностики АКТГ-продуцирующих опухолей.Примечание: AUC=0,888 (95% ДИ 0,774–1,000) для соматостатин-рецепторной сцинтиграфии, совмещенной с ОФЭКТ/КТ; AUC=0,870 (95% ДИ 0,796–0,944) для МСКТ с контрастным усилением.

Суммарные характеристики диагностических возможностей методов топической визуализации сведены в табл. 3. Необходимо отметить, что применение метода МРТ в верификации БИК в данной выборке пациентов (n=228) имеет чувствительность 60,2% (95% ДИ 52,6–67,5) и специфичность 59,2% (95% ДИ 44,2–73,0), а площадь под RОС-кривой составляет 0,597 (95% ДИ 0,507–0,687).

**Table:** Таблица 3. Диагностическая информативность методов топической диагностики в визуализации АКТГ-продуцирующих опухолей

	Диагностическая чувствительность, (95% ДИ)	Диагностическая специ­фичность, (95% ДИ)	Площадь под кривой (AUC)(95% ДИ)	Общая точность метода	Прогностическая ценность положительного результата	Прогностическая ценность отрицательного результата
МСКТ с контрастным усилением в установлении локализации НЭО	74% (59,7–85,4%)	100% (97,95–100%)	0,870 (0,796–0,944)	94,3% (90,1–96,9%)	100% (90,5–100%)	93,2% (88,6–96,3%)
Магнитно-резонансная томография головного мозга с в/в контрастированием в установлении БИК	60,2% (52,6–67,5%)	59,2% (44,2–73%)	0,597 (0,507–0,687)	60% (53,3–66,5%)	84,1% (76,6–90%)	29,3% (20,6–39,3%)
Сцинтиграфия с ОФЭКТ/КТ с 99mTc-Тектротидом в установлении локализации НЭО	73,3% (44,9–92,2%)	100% (95,3–100%)	0,888 (0,774–1,000)	95,7% (89,2–98,8%)	100% (71,5–100%)	95,1% (87,8–98,6%)

Примечание: МСКТ — мультиспиральная компьютерная томография; МРТ — магнитно-резонансная томография; ОФЭКТ/КТ — однофотонная эмиссионная компьютерная томография, совмещенная с компьютерной томографией; БИК — болезнь Иценко–Кушинга; НЭО — нейроэндокринная опухоль.

## ОБСУЖДЕНИЕ

В статье представлен обобщенный опыт проведения селективного забора крови из НКС с различными вариантами контроля положения катетеров, расчета АКТГ/пролактин-нормализованного отношения и методов топической визуализации на когорте пациентов с АКТГ-зависимым эндогенным гиперкортицизмом.

В Российской Федерации в связи с отсутствием кортиколиберина использовалась стимуляция десмопрессином. По результатам настоящего исследования показано повышение диагностических возможностей селективного забора крови из НКС, что подтверждено и в более раннем исследовании с меньшей статистической мощностью [13]. Сходные данные получены и в работах других авторов, где чувствительность данного метода в среднем превышала 90% [20–22]. В недавнем метаанализе 23 исследований, включивших в общей сложности 1642 пациента, чувствительность и специфичность двустороннего селективного забора крови из НКС составили 94% и 89% соответственно, что свидетельствует о высокой диагностической точности метода в дифференциальной диагностике БИК и АКТГ-эктопированного синдрома [23]. В целом чувствительность и специфичность процедуры колеблются от 88 до 100% и от 67 до 100% соответственно [24].

Важно отметить, что процедура должна выполняться только у пациентов с лабораторно подтвержденным АКТГ-зависимым эндогенным гиперкортицизмом на момент проведения селективного забора крови. Ремиссия гиперкортицизма, в том числе медикаментозная, делает результаты селективного забора неинформативными [12].

Контроль правильного положения катетера по градиенту пролактина позволяет исключить ложноотрицательный результат селективного забора крови. В нашем исследовании ложноотрицательные результаты в основном были связаны с аномальным оттоком крови из НКС и анатомическими особенностями каменистых синусов. В то же время необходимо отметить высокую результативность катетеризации, достигающую в общей сложности 97%. Определение градиента пролактина в ходе селективного забора крови из НКС является важным маркером забора крови именно от гипофиза.

Соотношение АКТГ с поправкой на пролактин предложено использовать для повышения точности метода и большей вероятности установки правильного диагноза. В проспективном исследовании впервые рассчитана отрезная точка 1,8 для АКТГ/пролактин-нормализованного отношения [14]. Предложенная отрезная точка имела некоторое преимущество в плане специфичности и, напротив, меньшую чувствительность по сравнению с традиционной оценкой градиента АКТГ (чувствительность 85,9% против 98,4%, специфичность 100% против 83,3% соответственно).

В нашем исследовании при сравнении диагностических возможностей селективного забора крови из НКС на фоне стимуляции десмопрессином с контролем положения катетера по градиенту пролактина и расчетом АКТГ/пролактин-нормализованного отношения продемонстрировано общее преимущество последнего (чувствительность 97,3% против 95,9%, специфичность 93,8% против 92% соответственно). Полученные данные в целом сопоставимы с результатами зарубежных исследований [25–27]. Определение градиента пролактина и одновременный расчет АКТГ/пролактин-нормализованного отношения позволяют увеличить диагностические возможности метода селективного забора крови из НКС на фоне стимуляции десмопрессином.

В качестве стандартных методов визуализации при топической диагностике АКТГ-продуцирующих опухолей в клинической практике широко применяют МРТ головного мозга в случае поиска АКТГ-секретирующей аденомы гипофиза и МСКТ внутренних органов при подозрении на АКТГ-эктопированный синдром. Однако изолированная информативность МРТ головного мозга в дифференциальной диагностике АКТГ-зависимых форм эндогенного гиперкортицизма является крайне низкой, и, как показало наше исследование, у 39,8% пациентов с АКТГ-продуцирующей аденомой гипофиза опухоль не визуализировалась при МРТ, в то время как у 40,8% больных с АКТГ-эктопированным синдромом по результатам МРТ выявлена гормонально-неактивная микроаденома гипофиза (инциденталома). При анализе диагностической информативности мы также получили крайне низкую чувствительность и специфичность метода МРТ в верификации БИК. Результаты нашей работы в целом согласуются с данными ретроспективного исследования Kaskarelis I. и соавт. [28], где также продемонстрирована недостаточная чувствительность МРТ в верификации пациентов с АКТГ-продуцирующей аденомой гипофиза в сравнении с двусторонним селективным забором крови из НКС (45,6% против 86,9%), но, в отличие от нашего исследования, более высокая специфичность МРТ (75% против 62,5%). Таким образом, низкая диагностическая ценность МРТ не позволяет использовать данный метод в качестве самостоятельного метода дифференциальной диагностики АКТГ-зависимого гиперкортицизма, и требуется исключение сочетания инциденталомы гипофиза и АКТГ-эктопированного синдрома при выявлении аденомы гипофиза менее 6 мм.

Гиперэкспрессия соматостатиновых рецепторов на клеточной мембране НЭО делает возможным проведение радионуклидных исследований с применением специальных РФП. Чувствительность и специфичность радионуклидной диагностики при АКТГ-продуцирующих НЭО значительно выше, чем при применении стандартных методов лучевой диагностики. Соматостатин-рецепторная сцинтиграфия является оптимальным методом радионуклидной визуализации НЭО, точность которой существенно повышается, если исследование выполняется с помощью гибридного метода, совмещающего однофотонную эмиссионную компьютерную томографию и рентгеновскую компьютерную томографию (ОФЭКТ/КТ), позволяющего дать полное представление об анатомическом расположении опухоли и рецепторной плотности [11]. С 2019 г. в Российской Федерации зарегистрирован РФП 99mTc-EDDA/HYNIC-TOC (99mTc-Тектротид) для визуализации образований с гиперэкспрессией соматостатиновых рецепторов (SSTR), имеющий наибольшую аффинность к SSTR2 в сравнении с SSTR3 и SSTR5. Результаты нашего исследования продемонстрировали высокую информативность гибридного метода радионуклидной диагностики — соматостатин-рецепторной сцинтиграфии с ОФЭКТ/КТ с 99mTc-Тектротидом, что во многих случаях стало основным критерием для выбора дальнейшей лечебной тактики. Диагностические возможности топической визуализации АКТГ-продуцирующих опухолей для соматостатин-рецепторной сцинтиграфии, совмещенной с ОФЭКТ/КТ, превосходили МСКТ с контрастным усилением по площади под ROC-кривой. Таким образом, у пациентов с установленным по селективному забору крови из НКС АКТГ-эктопированным синдромом оптимально проведение соматостатин-рецепторной сцинтиграфии, совмещенной с ОФЭКТ/КТ в качестве первой линии топической диагностики.

## Ограничения исследования

К ограничениям относятся ретроспективный анализ данных, следствием которого является отсутствие результатов некоторых исследований и пропуск в данных, а также малая выборка пациентов, которым проводилась соматостатин-рецепторная сцинтиграфия и ОФЭКТ/КТ. МРТ головного мозга выполнена на аппаратах с различной мощностью (1,5 и 3 Тесла), что могло повлиять на расчетную чувствительность и специфичность метода. Иммуногистохимическое исследование экспрессии КРГ рутинно не проводилось.

## ЗАКЛЮЧЕНИЕ

Усовершенствование метода двустороннего селективного забора крови из НКС путем оценки градиента пролактина, стимуляции десмопрессином и расчета АКТГ/пролактин-нормализованного отношения позволяет добиться улучшения диагностических возможностей метода. Для пациентов с установленным АКТГ-эктопированным синдромом согласно селективному забору крови из НКС оптимально проведение функциональных методов диагностики с применением меченных радиоактивным изотопом аналогов соматостатина, в частности соматостатин-рецепторной сцинтиграфии, что более информативно по сравнению с рутинным МСКТ с контрастным усилением. Менее инвазивные методы дифференциальной диагностики АКТГ-зависимого эндогенного гиперкортицизма с оценкой экспрессии микроРНК в периферической крови и протеомных маркеров могут быть предметом будущих исследований.

## ДОПОЛНИТЕЛЬНАЯ ИНФОРМАЦИЯ

Источники финансирования. Исследование проведено при поддержке Российского научного фонда (грант РНФ 19-15-00398-П).

Конфликт интересов. Авторы декларируют отсутствие явных и потенциальных конфликтов интересов, связанных с содержанием настоящей статьи.

Участие авторов. Голоунина О.О. — сбор и обработка полученных данных, формирование электронной базы данных, статистическая обработка, анализ полученных результатов, написание основного текста; Белая Ж.Е. — концепция и дизайн исследования, научное руководство проводимым исследованием, ведение пациентов, редактирование текста; Ситкин И.И. — выполнение селективного забора крови из нижних каменистых синусов, редактирование текста; Дегтярев М.В. — проведение радионуклидной диагностики пациентам, анализ полученных данных, редактирование текста; Лапшина А.М., Бондаренко Е.В. — патоморфологическое исследование удаленного опухолевого материала; Рожинская Л.Я., Трухина Д.А., Мамедова Е.О., Пржиялковская Е.Г. — ведение пациентов, редактирование текста; Мельниченко Г.А., Мокрышева Н.Г., Вакс В.В., Дедов И.И. — редактирование текста, одобрение финальной версии рукописи. Все авторы одобрили финальную версию статьи перед публикацией, выразили согласие нести ответственность за все аспекты работы, подразумевающую надлежащее изучение и решение вопросов, связанных с точностью или добросовестностью любой части работы.
